# Distinct double flower varieties in *Camellia japonica* exhibit both expansion and contraction of C-class gene expression

**DOI:** 10.1186/s12870-014-0288-1

**Published:** 2014-10-25

**Authors:** Yingkun Sun, Zhengqi Fan, Xinlei Li, Zhongchi Liu, Jiyuan Li, Hengfu Yin

**Affiliations:** Research Institute of Subtropical Forestry, Chinese Academy of Forestry, Fuyang, 311400 Zhejiang China; ᅟᅟ, Zhejiang Provincial Key Laboratory of Forest genetics and breeding, ᅟᅟ, ᅟᅟ Zhejiang China; College of Landscape Architecture and Forestry, Qingdao Agricultural University, Qingdao, 266109 Shandong China; Department of Cell Biology and Molecular Genetics, University of Maryland, College Park, ᅟᅟ Maryland USA

**Keywords:** Double flower, *AGAMOUS*, *Camellia*, Domestication

## Abstract

**Background:**

Double flower domestication is of great value in ornamental plants and presents an excellent system to study the mechanism of morphological alterations by human selection. The classic ABC model provides a genetic framework underlying the control of floral organ identity and organogenesis from which key regulators have been identified and evaluated in many plant species. Recent molecular studies have underscored the importance of C-class homeotic genes, whose functional attenuation contributed to the floral diversity in various species. Cultivated *Camellia japonica* L. possesses several types of double flowers, however the molecular mechanism underlying their floral morphological diversification remains unclear.

**Results:**

In this study, we cloned the C-class orthologous gene *CjAG* in *C. japonica*. We analyzed the expression patterns of *CjAG* in wild *C. japonica*, and performed ectopic expression in *Arabidopsis*. These results revealed that *CjAG* shared conserved C-class function that controls stamen and carpel development. Further we analyzed the expression pattern of *CjAG* in two different *C. japonica* double-flower varieties, ‘Shibaxueshi’ and ‘Jinpanlizhi’, and showed that expression of *CjAG* was highly contracted in ‘Shibaxueshi’ but expanded in inner petals of ‘Jinpanlizhi’. Moreover, detailed expression analyses of B- and C-class genes have uncovered differential patterns of B-class genes in the inner organs of ‘Jinpanlizhi’.

**Conclusions:**

These results demonstrated that the contraction and expansion of *CjAG* expression were associated with the formation of different types of double flowers. Our studies have manifested two different trajectories of double flower domestication regarding the C-class gene expression in *C. japonica*.

**Electronic supplementary material:**

The online version of this article (doi:10.1186/s12870-014-0288-1) contains supplementary material, which is available to authorized users.

## Background

Plant breeding is a process of human selection, which results in more desirable traits due to genetic modifications of key genes controlling plant development [1,2]. Several excellent examples have been reported in which key regulatory genes underwent human selection that led to alterations of gene function or expression resulting in desirable traits [3,4]. For instance, *Teosinte branched1* (*tb1*) of maize, encoding a TCP transcription factor, has been identified as a major contributor of branching changes in maize from its wild progenitor, *teosinte,* due to changes in its regulatory elements [3,5]. It is recognized that studies on the molecular genetic mechanism of plant domestication can provide valuable information to facilitate the modern genetic engineering, as well as illuminate the evolution of morphological adaptations [1].

The ABC model of flower development was initially established by genetic studies in *Arabidopsis thaliana* and *Antirrhinum majus* [6,7]. Three classes of floral organ identity genes, namely A B C, all encode MIKC^C^-type MADS-domain transcription factors except *APETALA 2 (AP2),* a class A gene coding for an AP2 domain transcription factor [6,8,9]. Both *A. thaliana* and *A. majus* bear canonical floral structure-the first whorl of sepals, second whorl of petals, third whorl of stamens, and carpels in the fourth and center whorl. According to ABC model, A- function genes specify sepals, B and A together specify petals, B and C together specify stamens, and C alone specifies carpels [6,9]. The following studies have elaborated this model to ABC(DE) in which D function controls ovule development and E function (*SEP*, *SEPALLATA* family genes) encodes co-factors of A, B, and C floral organ identity genes [10-12]. It is much clear in recent years that ‘A function’ might be only specific to *Brassicaceae* family, and the remaining features of the model seem widely conserved among flowering plants [12-14].

Nevertheless, the striking diversity of floral morphologies in different species suggests that evolutionary modifications of the A, B, and C gene functions may underlie the floral diversity. More and more characterizations in ‘non-model’ flowering species have reinforced the idea that non-canonical floral structures were often evolved by shifting expression or neo-functionalization of regulatory genes identified in model species [15,16]. For example, the inside-out floral organ arrangement in *Lacandonia schismatica* was in agreement with the altered expression of B- and C- function orthologs [17]. Similarly, functional elaborations of B-class genes in *Aquilegia* have been shown to contribute to the development of distinctive petaloid organs [18]. More surprisingly, despite markedly petaloid shape, the late expression of C- function gene was detected in the corona of daffodil [19], which suggested that corona might have a stamen-like origin but with changes of developmental pathways that dictating morphogenesis [19]. *AGAMOUS* (*AG*) is the only C class gene in *Arabidopsis* and its function in many higher plants including monocots are highly conserved [20,21]. In *Davidia involucrata*, the bract organ resembled petals, yet expressions of both B- and C- function homologs were detected [22], suggesting that certain expression combinations of ABC genes may not be sufficient to specify expected floral organ identities. The morphological innovations may require complex interactions of different genetic pathways or re-organization of gene expression levels during from initial pattern formation to organogenesis.

Double flower, characterized by excessive development of petals, is one of the most important traits of ornamental flowering species. Human selection over aesthetic traits is thought to play pivotal roles in the existence of vast variety of cultivated double flowers [2,4]. Recently the domestications of double flowers in some ornamental species have been recognized. In most cases, the double-flower varieties were derived from their wild ancestors bearing the single-flower [23,24]. Based on the framework of ABC model, in-depth investigations of the mechanism of double flower formation were carried out in many species [1]. In agreement with ABC model, loss of C function or expression modifications of the C function genes played a central role in the production of excessive numbers of petals. For example, in *Thalictrum thalictroides*, loss of function of the *AG* ortholog (*ThtAG1*) led to double flower development [25]. Also a mutation in the exon of *AG* homolog in *Prunus lannesiana* was found to lead to the formation of double flowers in this species [24]. In cultivated rose, restricted expression of *AG* orholog has been shown to contribute to the double flower development [1,26]. These studies, in essence, supported the basic tenet of the ABC model and revealed that manipulations of C class genes were critical for the domestication of double flowers in ornamental flowering plants. However, the molecular mechanism controlling different types of double flower forms remains elusive. The question of how human selection generates such a variety of double flower forms in a single species still remains unanswered. In *C. japonica*, like most other ornamental flowers, domestication process has resulted in several types of double flowers characterized by varying degree and morphology of excessive petals [27-29]. Five major types of double flower have been well documented regarding their distinctive arrangements of floral pattern, which suggested possibly multiple processes during which double flower domestication occurred. Among these double flower forms, the ‘anemone’ type is special due to distinct shapes of outer and inner petals, whilst typical double form displays a gradient changes of petal size [27,29]. Thus cultivated *C. japonica* may provide a unique system for studying the underlying mechanisms of double flower development as well as domestication. In this study, we identified the C-function otholog, *CjAG*, from *C. japonica*. Gene expression analysis and ectopic expression in transgenic *Arabidopsis* supported the conserved C-class function of *CjAG* in determining the stamen and carpel identities. We examined the expression patterns of *CjAG* in two different double flower varieties. In variety “Shibaxueshi” which lacked the stamen and carpel organs completely, the expression level of *CjAG* was significantly reduced or barely detected. In variety “Jinpanlizhi” which produced special inner petals, stamens and carpels in the center of flower, the expression level was detected in all the inner floral organs. Further analyses of expression patterns of B- and C- class genes in ‘Jinpanlizhi’ suggested that the morphological alterations of outer and inner petals were related to changes of gene expression levels during organogenesis. Our results revealed two different regulatory modifications of C-class gene expression in *C. japonica* during double flower domestication.

## Results

### Identification and sequence analysis of C-function gene in *C. japonica*

In order to identify the C-class gene in wild *C. japonica*, we designed degenerate primers based on alignment of different *AG* homologs from several plant species (Additional file 1: Table S1). Amplification products of homology cloning were sequenced and used to design gene specific primers for rapid amplification cDNA end (RACE) cloning (primers listed in Additional file 1: Table S1). Full-length sequence of *CjAG* was identified by assembly of different sequencing products and deposited in Genbank (Accession number: KM027370). The deduced protein sequence of *CjAG* was used to search for closest homologs against different plant species, and according to the result (not shown), *CjAG* was shown to be a member of *AG* family of MADS-box genes.

**Figure 1:**
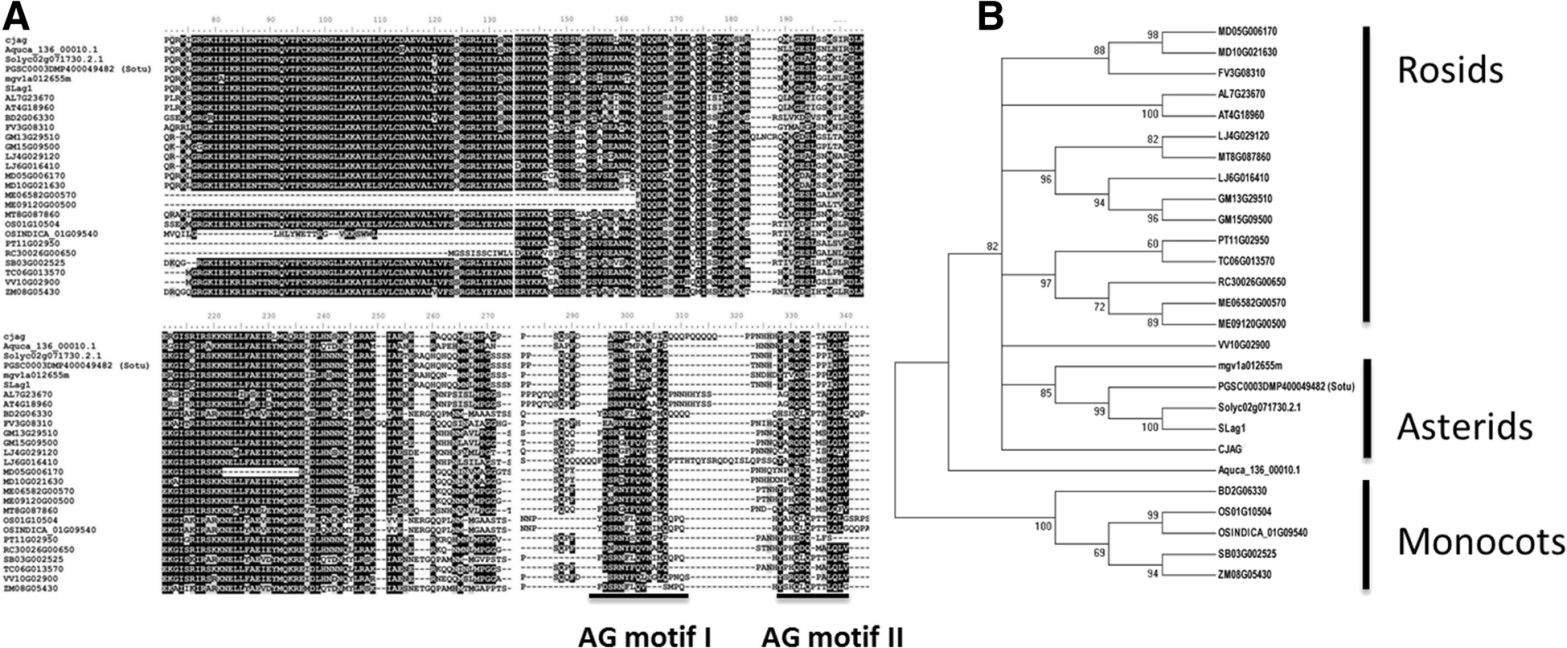
**Sequence alignment and phylogenic analysis of** ***CjAG.*** **A**, alignment of conserved regions of CjAG and related C- function orthologs. Two AG motifs were highlighted by underlines (Kramer [21]). **B**, a phylogenetic tree containing CjAG and other C- function othologs. Sequence information was listed in Additional file 2: Table S2.

To further characterize the phylogenetic relationships relevant to *CjAG*, we retrieved 26 othologous sequences of *AG* from 23 plant species as described in PLAZA 2.5 and other databases (Additional file 2: Table S2) [30]. We found that *CjAG* was highly conserved among all selected AG family orthologs by sequence alignment analysis (Figure 1A), and two *AG* motifs located at the C-terminal regions were also identified (Figure 1A) which supported that *CjAG* was an ortholog of *AG* in *C. japonica*. A phylogenetic tree was constructed by using those orthologous sequences (Figure 1B). We found that *CjAG* was placed within the core eudicot clade which was between *Vitis vinifera* and the asterid clade (Figure 1B). This result in parallel supported the origin of *CjAG* tracing back to *AG* common ancestor. Genus *Camellia* belongs to an order (Ericales) of clade asterids, and the placement of *CjAG* in the phylogenetic tree correlated well with its phylogeny.

### Ectopic expression of *CjAG* in *Arabidopsis*

**Figure 2:**
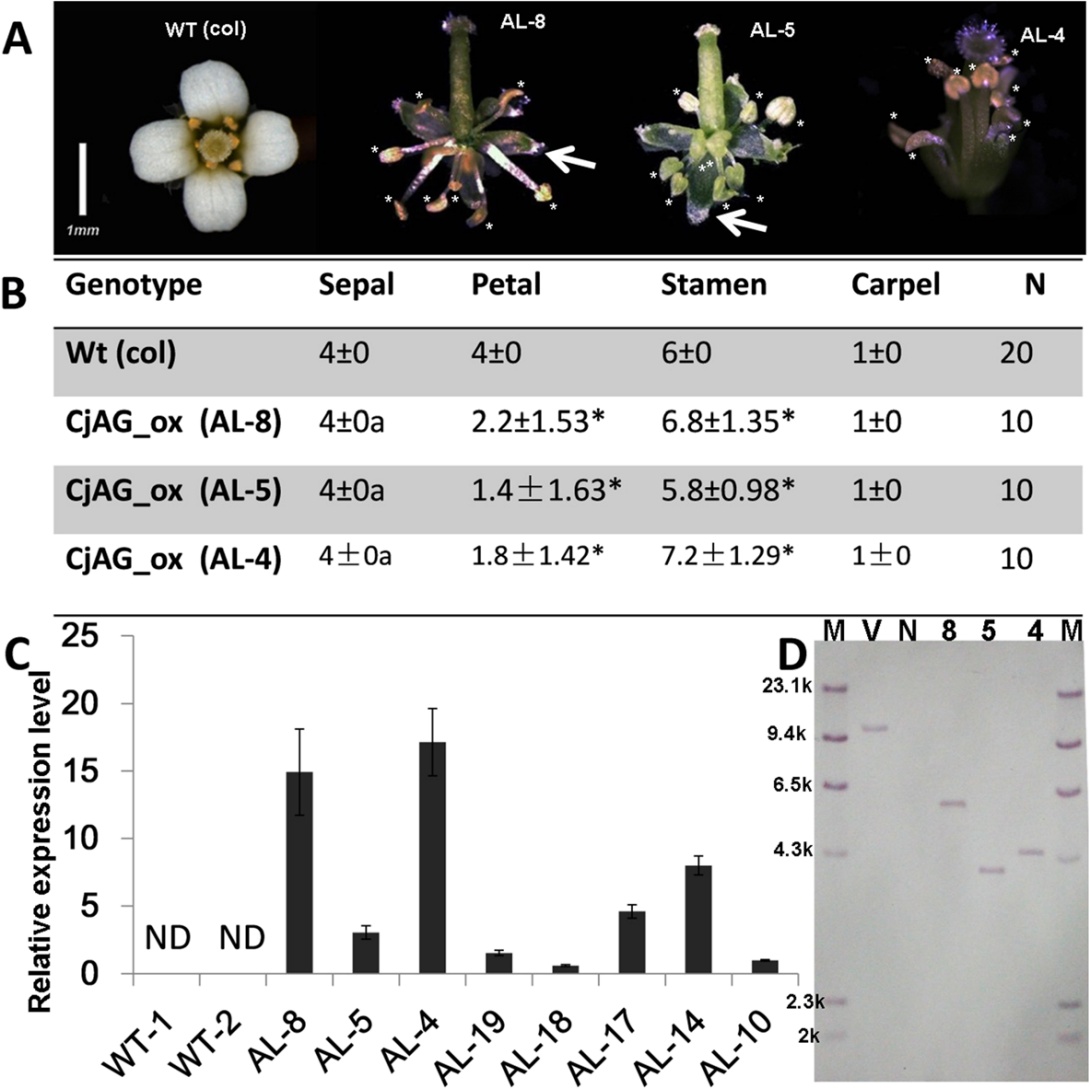
**Overexpression of** ***CjAG*** **in** ***A. thaliana*** **. A**, phenotypes of wt (columbia) and transgeneic plants. Overexpression plants displayed no or less petal development, and increased the number of stamens. White stars indicated stamens. **B**, statistical analysis of floral organ numbers in wt and transgenic plants. a, indicated abnormal morphologies of sepals in transgenic plants. Stars indicated p <0.05 by student’s t-test comparing to wt. **C**, expression levels of *CjAG* in 8 independent transgenic lines. ND not detectable. **D**, three lines were verified as single insertion events by southern blotting. Arrows indicated pistil-like structures observed in sepals of transgenic plants. M, maker; V, vector control; N, negative control; 8, line AL-8; 5, line AL-5; 4, line AL-4.

The C-class genes have been found to possess highly conserved functions of determining stamen and pistil identity in many eudicot species. To address whether *CjAG* has similar functions in floral patterning to other species, we generated transgenic *A. thaliana* with ectopic expression of *CjAG*. The construct was driven by the cauliflower mosaic virus (CaMV) 35S promoter, and transformed into wild type (wt) *A. thaliana* through agrobacterium mediated transformation [29]. We screened and identified positive lines by selectable marker tests and PCR analysis with construct-specific primers (Additional file 1: Table S1). Eight positive lines (AL-8, AL-5, AL-4, AL-19, AL-18, AL-17, AL-14, AL-10) were identified and selected for further expression analysis (Figure 2C). Three potential single-insertion T2 lines were identified by genetic segregation analysis, and were tested by southern blotting analysis (Figure 2D). Three T2 lines (AL-4, AL-5, AL-8) shown single insertion by southern blotting were further characterized for phenotypic analysis (Figure 2A-B). To access the level of ectopic expression of target gene, the qRT-PCR experiment using gene-specific primers was performed in selected transgenic lines, and increased expression levels of *CjAG* in *Arabidopsis* were detected (Figure 2C). The three lines AL-4, AL-5, AL-8 displayed about 16, 14 and 4 folds of expression comparing to the lowest line AL-18 (Figure 2C) respectively.

All three (AL-8, AL-5, and AL-4) lines of transgenic plants displayed abnormal development of flowers when compared with non-transgenic wt *Arabidopsis*. Petals were partially or entirely absent, and the number of stamens was increased (Figure 2A-B). Detailed statistical analysis revealed that the number of petals was significantly reduced, and number of stamens was significantly increased when compared with wt (Figure 2B). The number of sepals remained the same as wt, the 35S::CjAG transgenic plants developed abnormal sepals with pistil-like features including stigma (Figure 2A). Interestingly, the transgenic plants did not develop extra carpels (Figure 2A-B). Since C function is known to antagonize A function genes and ectopic expression of C function in *Arabidopsis* led to conversion of sepals to carpels, and petals to be absent or converted to stamens [31], our data supported that *CjAG* possessed the conserved C-class function due to a similar but a weaker effect. The weaker effect could be explained by *CjAG*’s functioning in a heterologous system.

### Comparisons of single and double flower patterns in *C. japonica*

**Figure 3:**
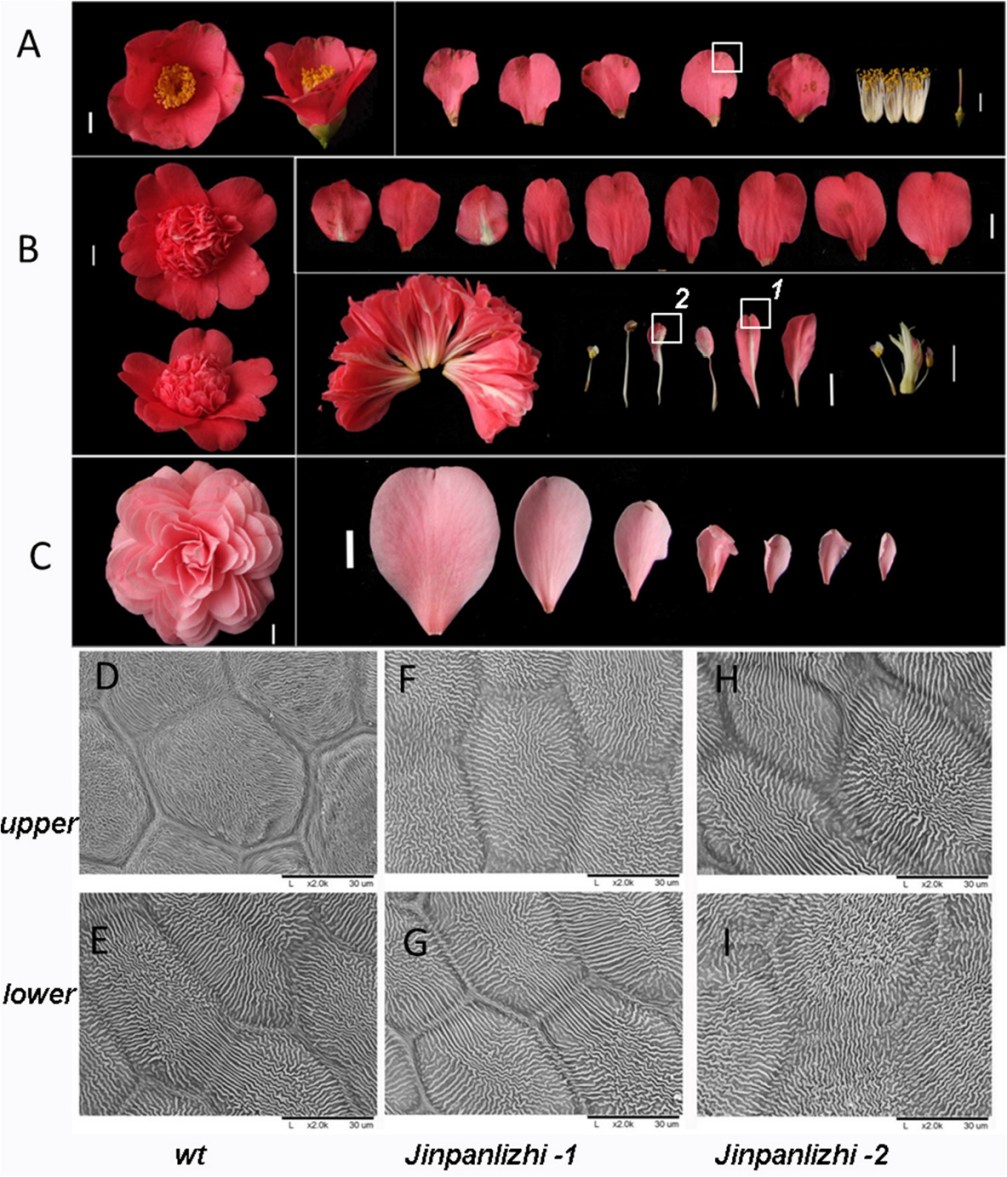
**Comparison of floral patterns in wild and cultivated camellias. A**, wild *C. japonica* was singe-flower with canonical floral structures. **B**, double-flower cultivar ‘Jinpanlizhi’ displayed distinctive shapes between outer and inner petals. Right upper panel of B displayed the outer petals from outside to inside; Right bottom panel showed the inner organs including inner petals, stamens, carpels, and stamenoid petals. **C**, double-flower cultivar ‘Shibaxueshi’ was a typical formal double type with gradient petals from outer layer to the inside. The stamen and carpel were missing. **D**, **E**, upper and lower epidermal cells from wt; **F**, **G**, upper and lower epidermal cells from ‘Jinpanlizhi’; **H**, **I**, upper and lower epidermal cells from ‘Jinpanlizhi’. White squares indicated the areas used for SEM analysis, and 1 and 2 were referring to **F**, **G** and **H**, I respectively.

The wild single flower of *C. japonica* displayed canonical floral structures which consisted of sepal, petal, stamen and pistil. In most occasions, a single whorl of 5 to 6 petals is found in wild *C. japonica* (Figure 3A). ‘Jinpanlizhi’ and ‘Shibaxueshi’ were two popular double-flower cultivars in which both had multiple whorls of petals and retarded or missing reproductive organs (Figure 3A-C). However, the petal patterns of ‘Jinpanlizhi’ and ‘Shibaxueshi’ differed distinctively. ‘Jinpanlizhi’ was a typical anemone type of double flower, in which two distinct layers of petals were formed (Figure 3B). The outer layer of petals morphologically resembles petals of single flower, and 9–11 petals are usually found in 2–3 overlapping whorls (Figure 3B). The inner area consisted of a large number of petal-like organs, and some of them were typical mosaic organs of petal and stamen (Additional file 3: Table S3; Figure 3B). Detailed morphological dissections revealed that inner petals were different from outer petals in shape. The gradient changes from stamens to petaloid stamens to inner petals suggested that inner petals might partially acquire petal identity through conversion of stamens. But the total floral organ number was increased comparing to wt (Additional file 3: Table S3). In order to address this further, we performed Scanning Electron Microscopy (SEM) analysis to check the morphological characteristics of petals epidermal cells in wild petals and inner petals of ‘Jinpanlizhi’. We showed that in most expanded area, both sides of wild and ‘Jinpanlizhi’ petals had flat epidermal cells in which rugose textures were found (Figure 3D-I). Despite the marked change in shape, inner petals of ‘Jinpanlizhi’ had similar epidermal cells with wild single-flower petals. The ‘Shibaxueshi’ cultivar is a typical formal double flower variety in which stamens and pistils were completely missing and replaced by petals (Figure 3C), and the gradient changes of petal shape were also seen (Figure 3C).

### Expression of *CjAG* displayed different patterns between ‘Jinpanlizhi’ and ‘Shibaxueshi’

**Figure 4:**
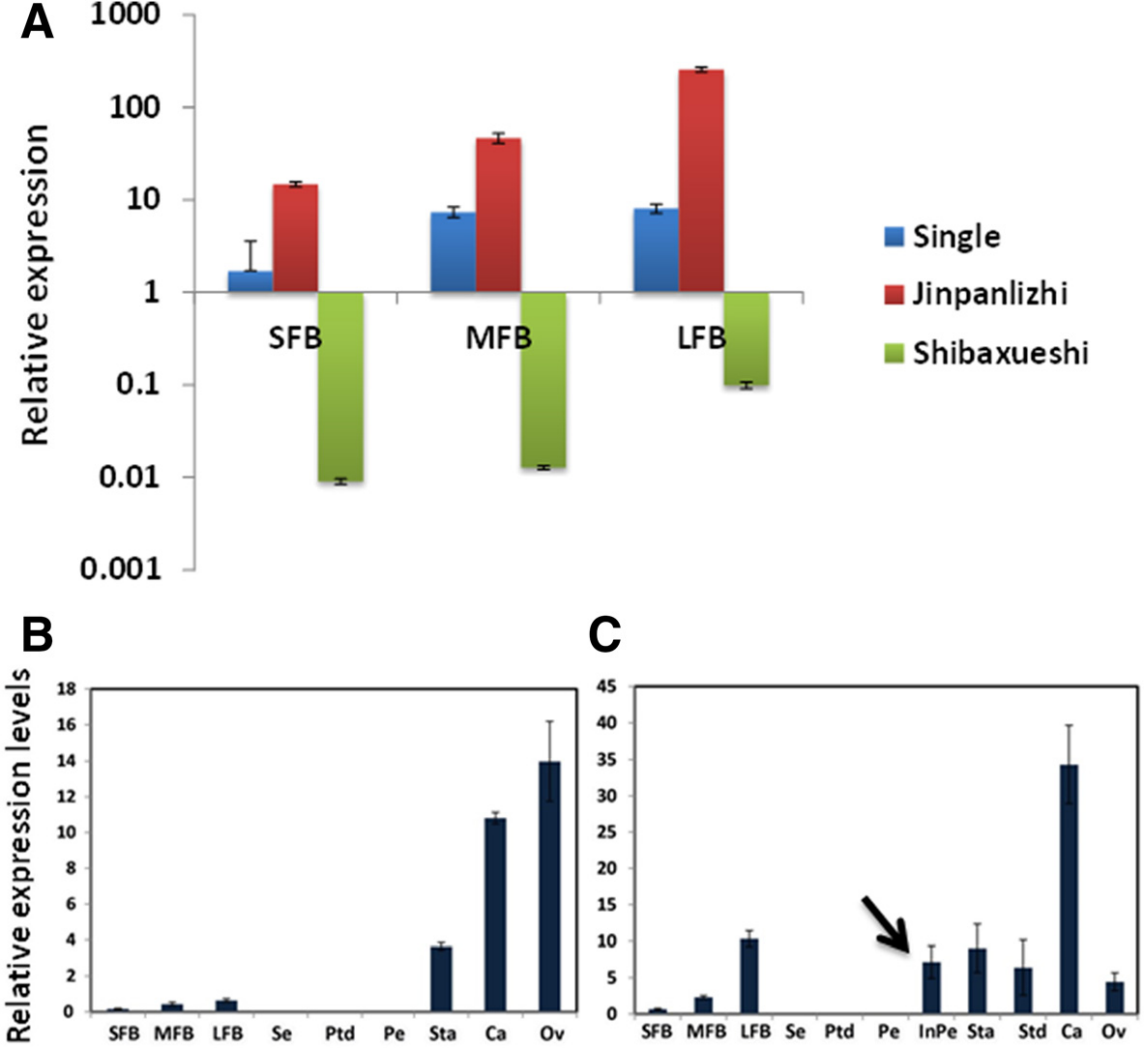
**Expression analysis of** ***CjAG.*** **A**, Expression levels of *CjAG* in three developmental stages of floral buds of wt, ‘Jinpanlizhi’ and ‘Shibaxueshi’. **B**, expression of *CjAG* in different floral organs in wt. **C**, expression of *CjAG* in different floral organs in ‘Jinpanlizhi’. SFB, early stage of floral bud initiation; MFB, floral organ initiation; LFB, floral bud outgrowth; Se, Sepal; Ptd, Petaloid sepal; Pe, Petal; Sta, Stamen; Std, Stamenoid petal; Ca, Carpel; Ov, Ovule. Arrow indicated expression of *CjAG* in inner petals.

In consideration of the classic ABC model, we were asking whether the modification of C-class gene was involved in the formation of double flower in ‘Jinpanlizhi’ and ‘Shibaxueshi’. Firstly we identified the full-length coding sequences of *CjAG* from ‘Jinpanlizhi’ and ‘Shibaxueshi’, and we found there were no coding sequence changes in neither of the two varieties (not shown). Further, we compared the expression levels of *CjAG* between different developmental stages of floral bud (Figure 4A). Surprisingly, we found that the expression levels of *CjAG* in ‘Jinpanlizhi’ and ‘Shibaxueshi’ displayed different patterns comparing to wt (Figure 4A). In ‘Shibaxueshi’ the expression levels of *CjAG* at all three staged [SFB, early stage of floral bud initiation (1-3 mm); MFB, floral organ initiation (4-8 mm); LFB, floral bud outgrowth (9-13 mm)] were remarkably reduced (Figure 4A), which suggesting a loss of C-class gene expression was involved in double flower development. Nevertheless, the expression levels of *CjAG* in ‘Jinpanlizhi’ were significantly increased when compared with the wt (Figure 4A). To investigate how the increased expression of *CjAG* occurred in ‘Jinpanlizhi’ we examined the expression levels of *CjAG* in different floral organs. We found that the expression of *CjAG* in wt was detected in stamens and carpels, but not in sepals and petals, which was expected for C-class genes (Figure 4B); In ‘Jinpanlizhi’, the expression of *CjAG* was not only detected in inner stamen, petaloid stamen and carpel like organs, but also in inner petals. No expression was identified in outer petals (Figure 4C).

**Figure 5:**
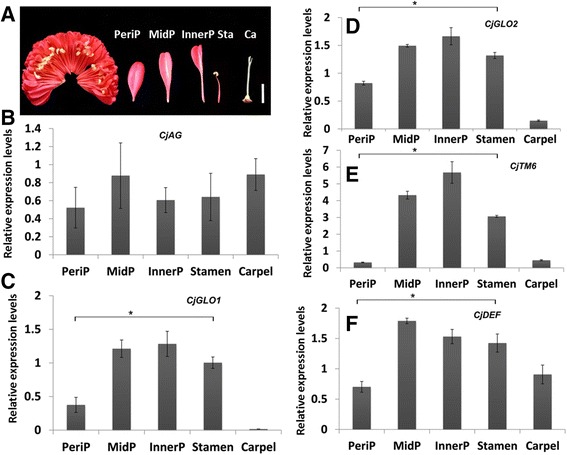
**Expression analysis of B- and C- class genes in the inner organs of ‘Jinpanlizhi’. A**, typical organs used for expression analysis. **B**, Expression levels of *CjAG* in different inner organs of ‘Jinpanlizhi’. **C**, Expression levels of *CjGLO1* in different inner organs of ‘Jinpanlizhi’. **D**, Expression levels of *CjGLO2* in different inner organs of ‘Jinpanlizhi’. **E**, Expression levels of *CjTM6* in different inner organs of ‘Jinpanlizhi’. **F**, Expression levels of *CjDEF* in different inner organs of ‘Jinpanlizhi’. PeriP, Periphary petal; MidP, Middle petal; InnerP, Inner petal; Sta, Stamen; Ca, Carpel. Stars indicated p <0.05 by student’s t-test.

The shapes of inner petals varied gradually from oval to filamentous-like in ‘Jinpanlizhi’ (Figure 5A). The expression of B-class genes were thought to be critical for the petal evolution and development, but the co-expression with C-class gene determine the stamen organ identity. In *C. japonica,* B-class genes underwent recent duplications and were expressed in petals and stamens, as well as carpels [32]. To explore how B- and C-class genes behavior in inner organs, we checked expression patterns of four B- class and *CjAG* in different types of inner organs (Figure 5B-F). We showed that *CjAG* was expressed in all inner organs with similar expression levels (Figure 5B), and B-class genes (*CjGLO1/2*, *CjTM6, CjDEF*) had differential expression levels between different inner organs, but only the periphery inner petals displayed significantly lower expressions than stamens (Figure 5C-F). Considering the lack of *CjAG* expression in outer petals, these results indicated the differential expression levels of B- and C- class gene might contribute to the inner organ morphogenesis.

## Discussion

### Multiple trajectories of double flowers domestication in *C. japonica*

Double flower is potentially the most important traits of ornamental flower species, and in many commercial flowers single flower is of no or low market values [23,33,34]. According to the studies of *AG* in *Arabidopsis*, the C-class gene not only determined the stamen and carpel identities, but also controlled the determinacy of inflorescences [35]. Thus attenuated C-class function could increase petal development, inhibit stamen development, and increase floral organ number as well, which perfectly predicts the formation of double flower [36].

Current studies in various ornamental plants have revealed that many double flower domestications were related to the modification of C-class functions [1,25,26]. However, unlike the case of ‘Jinpanlizhi’, these events caused either loss or reduce of C-class gene function. Therefore to study how expansion of C-class gene expression is related to double flower formation is not only important to help the genetic improvement of new ornamental traits, but also presents an opportunity to address the mechanism of phenotypic adaptations. Particularly, the domestication of double flower in *Camellia* and other related species has resulted in different types of double flower patterns [27,33]. Notably, five major types of double flower were identified by morphological characterizations of flower organ number, organ shape and compositions [27,28], which suggested various diversifications of molecular mechanisms underlying the control of double flower development. The ABC model has set up a genetic model of floral organ identity determination in which A- and B-class genes together controlled petal development, while later studies in other higher plants suggested petal evolution and development was regulated by B-class genes [8,37,38], and A- function might be species specific [37]. As it has been shown, *AP1/FUL* like genes in *C. japonica* appear to be related to double flower formation by increasing their expression levels, suggesting A- and C- types genes were both modified during double flower development [31]. Studies in several ornamental flower species have revealed that C-class genes were responsible for the formation of double flowers [1]. Either lost or reduced expression of C- class genes would increase petal development and inhibit stamen development, which, in essence, was coinciding with classic ABC model. Indeed, Lenser and Theissen have reviewed current studies and pointed that C-class gene *AG* was a ‘nodal’ factor regarding double flower [1,26].

As the case in *C. japonica*, no mutations in the coding region of *CjAG* have been found in different types of double flower varieties. In ‘Shibaxueshi’, the expression of *CjAG* was barely detectable, which might explain the formation of formal double flowers (Figure 4A). Expression analysis in other types of double flowers apparently indicated a more complex scenario of alterations of *CjAG* expression. In variety ‘Jinpanlizhi’ the expression levels of *CjAG* were up-regulated in inner organs including petals, petaloid stamens and carpels, while no expression was detected in outer petals (Figure 4B-C). The distinctive shapes of outer and inner petals indicated that *CjAG* was potentially involved in the inner petal development (Figure 3). Recent findings in *Narcissus bulbocodium* and *Davidia involucrata* have revealed an unexpected expression of C-class genes in bract and corona – like organs, and these organs were uncanonical organs referring to ABC model [19,22]. So, to understand the divergent roles of C-class genes in plant species requires extensive functional analysis in non-model species. Although it is not clear at this point whether a post-transcriptional regulation is evolved specifically, the diversification of regulatory pathways regarding organ development is evident. The various types of double flowers in *C. japonica* present a system to study how domestication could impact floral development pathways to generate new floral traits. The comparison of *CjAG* expression in ‘Shibaxueshi’ and ‘Jinpanlizhi’ suggests that C-class gene is an important target of double flower domestication; however, multiple trajectories are involved in tuning the expression pattern of *CjAG*. The sequence changes at the regulatory regions of *CjAG* might be critical for altering the expression patterns in both ‘Jinpanlizhi’ and ‘Shibaxueshi’ cultivars. And it is possible that different mutations could be responsible for up- and down- regulations of *CjAG* expression in these double flowers. Further studies in the promoter and regulatory regions of *CjAG* are required to demonstrate how genetic modifications may affect *CjAG* expression.

### Petal organogenesis and ABC genes expression in ‘Jinpanlizhi’

It has been shown that B-class genes in *C. japonica* expressed in petals and stamens, and also with less levels in carpels [32]. Quantitative gene expression analysis in inner organs of ‘Jinpanlizhi’ has revealed that expression levels of B-class genes varied between inner petals, petaloid stamens and carpels, while *CjAG* expressed consistently in these organs (Figure 5B-F). These observations suggested *CjAG* might retain the expression domains in the floral meristem in ‘Jinpanlizhi’, but potentially the changes of other developmental regulators, such as GLO/DEF-like genes, played critical roles at the stage of petal organogenesis. As it is seen in *A. majus*, the late stage development of petal has been shown to be regulated by transcript levels of B- class genes (*DEF*, *GLO*) and other transcriptional regulators, and the autoregulation loops of these components were required for elaboration of petal development [39]. It is possible that at the early stage of development, C- class expression is not sufficient to dictate the organogenesis process to distinguish the petal and stamen specification; in ‘Jinpanlizhi’ the morphological changes of inner organs might rely on the modification of gene networks of petal outgrowth. Therefore, the involvement of *CjAG* in inner petal development could be a main factor of distinguishing it from outer petal morphogenesis. In consideration of AG- and PLE- lineages of C-class genes [40], another possibility is that the PLE- type gene may play important roles for defining the C- function in *Camellia*; also due to the lack of genome-wide analysis, it is not known whether duplication of ABC genes is involved in the double flower formation. Despite the fact that the functions of C- class genes have been examined extensively, in-depth analyses of *CjAG* and other floral regulators are still needed to further understand the mechanism of double flower formation under human selection.

## Conclusions

The domestication of double flower in many ornamental species has underscored the central roles of C-class function genes [1]. Contracted expression or loss-of-function mutations were revealed to contribute to the formation of excessive petals in various double flowers [1,24-26]. In this work, we isolated the *AG* ortholog gene, *CjAG*, from *C. japonica. CjAG* expressed predominantly in stamens and carpels in wild *C. japonica*, and ectopic expression of *CjAG* in *Arabidopsis* resulted in increased number of stamens and reduced petals. These results supported the conserved C-functions of *CjAG* in *C. japonica*.

Furthermore, we examined the expression patterns of *CjAG* in two double flower cultivars, ‘Shibaxueshi’ and ‘Jinpanlizhi’, which displayed different petal patterns. We found that the expression of *CjAG* was markedly down-regulated during floral development of ‘Shibaxueshi’; while up-regulated in ‘Jinpanlizhi’. Detailed expression analyses of *CjAG* in inner organs of ‘Jinpanlizhi’ revealed that *CjAG* expanded its expression in inner petals. Finally, expression profiling of B-class genes in ‘Jinpanlizhi’ suggested that considerable modulations of expression pattern of floral regulators might be involved in the organogenesis of inner petals.

In conclusion, we demonstrate that the alterations of *CjAG* expression were involved in the domestication of two types of double flowers in *C. japonica*. These results have revealed two different trajectories targeting the C-function gene during double flower formation in *C. japonica*.

## Methods

### Plant materials and growth conditions

Camellia materials used in this study were grown in the greenhouse of Research Institute of Subtropical Forestry located in Fuyang (119°57′N, 30°04′ E; Fuyang city, Zhejiang, China) under natural light condition. The annual mean temperature was about 18°C with regular irrigations. For collecting samples of RNA, healthy floral buds or organs at different developmental stages were collected and frozen immediately in liquid nitrogen and stored in −80°C freezers before use. *Arabidopsis* (Columbia) seeds were sterilized and grown on agar plates containing 1/2 Murashige and Skoog medium at 4°C for 2 days. The seedlings were then grown in growth chambers under long-day conditions (16 h light/8 h dark) at 22°C for 10 days before being transplanted to soil. The light intensity of the growth chambers was 150 mE m^−2^ s^−1^. All original materials were collected under the permission of local authorities, and voucher specimens were deposited in the Research Institute of Subtropical Forestry.

### Scanning electron microscopy analysis

Petal samples were collected by cutting into small pieces and fixed in FAA solution (formalin: glacial acetic acid: 70% ethanol = 1:1:18) as described [41]. The fixed samples were dehydrated by going through the gradual ethanol series, and then dried by critical point drying method by liquid carbon dioxide (Model HCP-2, Hitachi, Japan) and then gold-coated by an Edwards E-1010 ion sputter coater (Hitachi, Japan). The samples were observed with a S-3000 N variable pressure scanning electron microscope (Hitachi, Japan).

### Isolating *CjAG* in *C. japonica* and phylogeny analysis

Total RNA was extracted from floral buds by using the Column Plant RNAout2.0 kit and treated with Column DNA Erasol (Beijing Tiandz Gene Technology Company, Beijing, China) to avoid the DNA contamination. To generate RACE products, the purified total RNA was reverse transcribed by adapted primers according to the manufacturer’s instructions (Clontech, USA). Touchdown PCR was performed to amplify target genes by combining a degenerate primer and the adaptor primer (Clontech, USA). Multiple PCR products of gradient amplification (annealing temperature from 49°C to 62°C) were purified and cloned into pMD18-T easy vector (Takara, Dalian, China) for sequencing. Sequences were assembled by multiple fragments from RACE and full length open reading frame was confirmed by PCR amplification and sequencing. The sequence of *CjAG* was deposited in public database [GenBank: KM027370]. Primers are listed in Additional file 1: Table S1. Deduced protein sequences of *CjAG* was aligned with protein sequences of other AG othologous genes derived from PLAZA2.0 by clustalW [30]. Phylogenetic trees were made by MEGA5 using NJ method according to the manual [42].

### Quantitative PCR analysis

Total RNA was extracted and treated with DNAse as described [29]. The purified total RNA was reverse transcribed using oligo (dT) primer by PrimeScript RT reagent Kit (TAKARA, Japan). The gene-specific primers of PCR amplification for target genes were designed by Primer Express 2.0 (Applied Biosystems) and tested the amplification specificity before quantification experiment. The 18S rRNA was used as an internal control as described before [43]. The real-time PCR reaction was performed on an ABI PRISM 7300 Real-Time PCR System (USA) by using SYBR Premix Ex Taq (TAKARA, Japan). Amplification occurred in a two-step procedure: denaturation at 95°C for 30 s and followed 40 cycles with denaturation at 95°C for 5 s, 60°C for 31 s. After completion of the amplification steps, the melting curve was determined for each analysis and the data were analyzed with the 2^-ΔΔCT^ method [44].

### Transformation of Arabidopsis and analysis of transgenic plants

To generate overexpression vectors of *CjAG*, the full coding region was amplified by gene specific primers (Additional file 1: Table S1) and cloned into pMD18-T vector (Takara, Dalian, China). Plasmids containing correct sequences and right directions were identified by sequencing, and subsequently cloned into pCAMBIA1300_35S binary vector [29]. The plasmids were introduced *into Agrobacterium tumefaciens* GV3101 by heat shock method. *Agrobacterium tumefaciens* mediated transformation of *A. thaliana* was performed essentially as described [29] with minor modification. T1 seeds were placed on MS medium containing 50 mg/L Hyg and positive seedlings were transferred to pots and grown in a growth chamber. T1 and T2 seedlings were identified for further analysis. Images were obtained through a Leica MP6 dissecting microscope.

### Genomic DNA extraction and southern blotting

About 5 μg genomic DNA from three independent T2 transgenic lines was digested with restriction endonuclease EcoRI (MBI Fermentas, Canada) at 37°C for 16 hours, electrophoretically separated on a 1.2% agarose gel and transferred to a positively charged nylon membrane. The lambda DNA with digoxigenin labeling (Cat. 11218590910, Roche) was used as marker. The DNA was fixed on the membrane by baking at 120°C for 30 min. The preparation of probe, pre-hybridization, hybridization and immunological detection were all performed according to the protocol of DIG-High Prime DNA Labeling and Detection starter Kit (Roche, USA). The gene specific probes were amplified by using primers listed in Additional file 1: Table S1.

### Availability of supporting data

All the data supporting our results are included in the article and in the Additional files.

## Additional files

Additional file 1: Table S1. Primer list. Additional file 2: Table S2. Information of sequences used for phylogenic analysis. Additional file 3: Table S3. Counting of floral organs in wt and cultivar ‘Jinpanlizhi’.

## Abbreviations

RACE: rapid amplification cDNA end
SEM: Scanning Electron Microscopy
CaMV: cauliflower mosaic virus
wt: wild type
