# Supplementary figures and images for: Distinct double flower varieties in Camellia japonica exhibit both expansion and contraction of C-class gene expression
Source: BMC Plant Biol. 2014 Oct 25;14:288. doi: 10.1186/s12870-014-0288-1 (PMC4219040; doi:10.1186/s12870-014-0288-1)

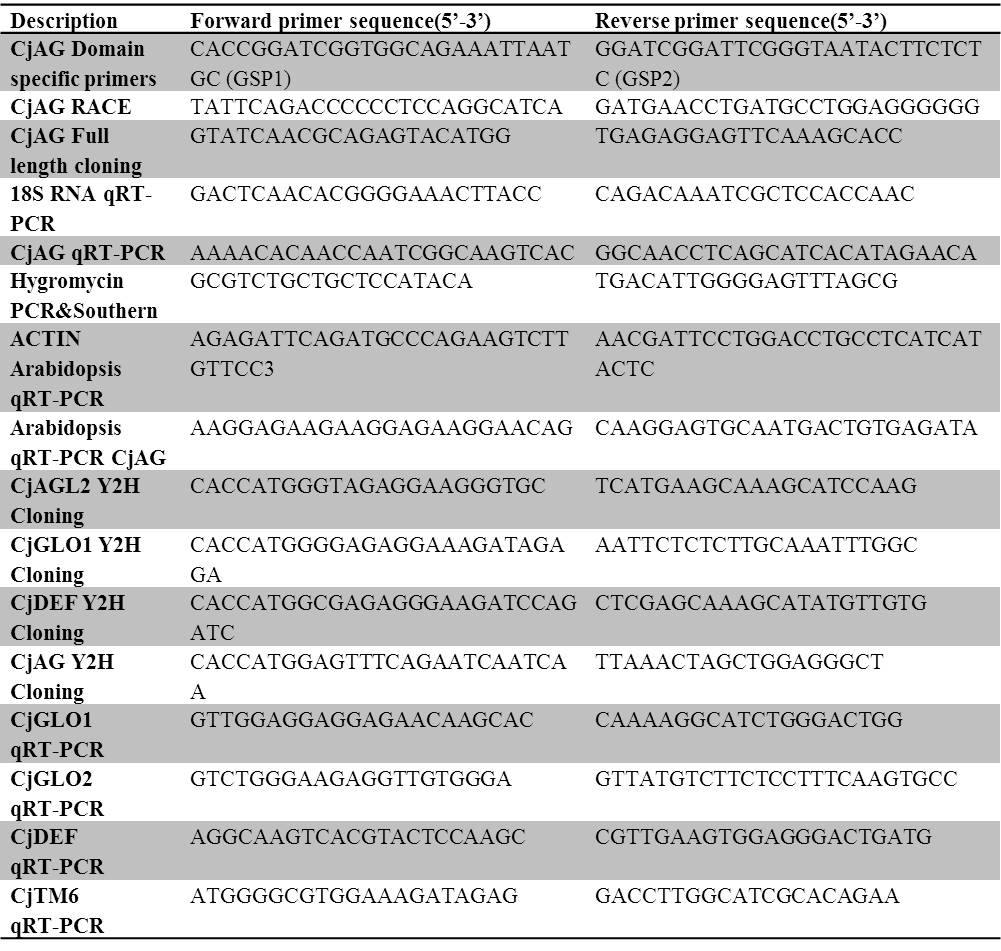

Supplement: Additional file 1: Table S1. — Primer list. [file 12870_2014_288_MOESM1_ESM.jpeg]

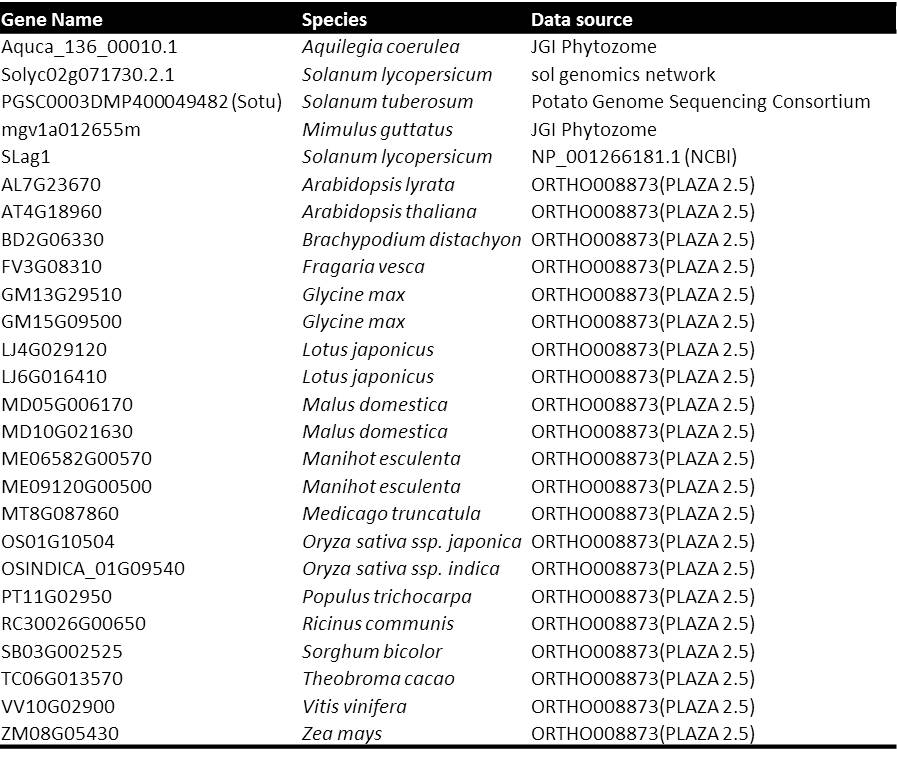

Supplement: Additional file 2: Table S2. — Information of sequences used for phylogenic analysis. [file 12870_2014_288_MOESM2_ESM.jpeg]

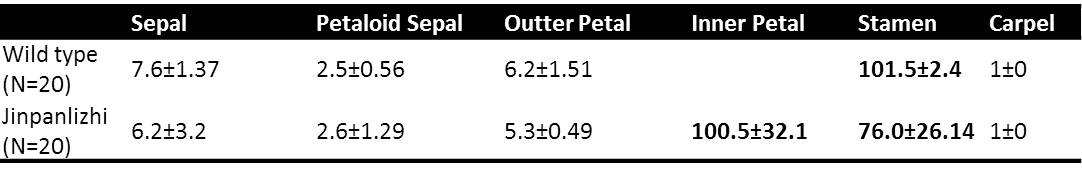

Supplement: Additional file 3: Table S3. — Counting of floral organs in wt and cultivar ‘Jinpanlizhi’. [file 12870_2014_288_MOESM3_ESM.jpeg]
